# Reduced mitochondrial function in obesity-associated fatty liver: SIRT3 takes on the fat

**DOI:** 10.18632/aging.100289

**Published:** 2011-02-26

**Authors:** Mahua Choudhury, Karen R. Jonscher, Jacob E. Friedman

**Affiliations:** ^1^ Department of Pediatrics, University of Colorado School of Medicine, Aurora, CO 80045, USA; ^2^ Department of Anesthesiology, University of Colorado School of Medicine, Aurora, CO 80045, USA; ^3^ Department of Biochemistry and Molecular Genetics, University of Colorado School of Medicine, Aurora, CO 80045, USA

**Keywords:** aging, metabolism, obesity, SIRT3, mitochondria

## Abstract

Aging is associated with various metabolic disorders that may have their origin in the liver, including non-alcoholic fatty liver disease, obesity, type 2 diabetes mellitus, and atherosclerosis. Although well-characterized in models of caloric restriction, relatively little is known about the role of sirtuins and acetylation under conditions of caloric excess. Sirtuins are NAD (+)-dependent protein deacetylases that mediate adaptive responses to a variety of stresses, including calorie restriction and metabolic stress. Sirtuin 3 (SIRT3) is localized within the mitochondrial matrix, where it regulates acetylation levels of a diverse set of metabolic enzymes. When normal mice are fed a high fat diet they demonstrate reduced SIRT3 activity, impaired mitochondrial function, and hyperacetylation of a diverse set of proteins in their livers. Furthermore, SIRT3 knockout mice have signs of accelerated aging and cancer. Understanding SIRT3's biochemical function and regulation in the liver under conditions of caloric excess may potentially increase our understanding of the normal aging process and diseases associated with aging, such as diabetes, fatty liver disease, or cancer.

Aging is associated with various metabolic disorders that may have their origin in the liver, including obesity, type 2 diabetes mellitus, non-alcoholic fatty liver disease (NAFLD), and atherosclerosis [1,2]. These conditions are provoked by diverse factors, including reactive oxygen species, endoplasmic reticulum stress, hypoxia, lipotoxicity, and altered adipokine signaling [3-5]. In addition, saturated fatty acids, which are increased in obesity [5-8], have been implicated in the coordinate regulation of metabolism with inflammatory and immune responses in the liver [9-11].

The mitochondrial proteome also changes with disease state. Recently, mitochondrial dysfunction has been implicated in the pathology of chronic metabolic disease characterized by insulin resistance such as obesity, type 2 diabetes mellitus, and aging. Acetylation has emerged as an important mechanism for controlling metabolism of a broad array of metabolic fuels. Acetylation regulates many enzymes in key pathways including TCA cycle, gluconeogenesis, and beta oxidation in yeast and human liver [12,13]. This post-translational modification is governed, in part, by sirtuins, class III NAD+-dependent deacetylases (HDACs) that regulate lipid and glucose metabolism in liver during fasting and aging. SIRT3, a mitochondrial sirtuin, appears to be the primary mediator of mitochondrial acetylation, since no significant changes in acetylation status are detectable in mice lacking SIRT4 and SIRT5 [14].

## Mice lacking SIRT3 show accelerated aging and liver phenotypes during fasting

All sirtuins require NAD+ for their deacetylase activities, linking their functions as metabolic sensors. For example, increased Nampt-mediated NAD biosynthesis enhances SIRT1 activity in mouse fibroblasts [15]. Nampt also plays an important part in regulating cellular stress resistance through SIRT3 [16]. Under genotoxic stress, increased Nampt plays an important part in maintaining NAD levels in mitochondria and providing protection against cell death by suppressing translocation of apoptosis-inducing factor from mitochondria to the nucleus [16]. These protective effects of Nampt require mitochondrial SIRT3 [17].

Previously it was shown that many mitochondrial proteins are hyperacetylated in SIRT3 knockout mice on a standard chow diet; however SIRT3-/- mice were only mildly distinguishable from WT littermates [14]. As they aged however, 13 month old SIRT3 knockout mice showed accelerated signs of aging in the heart, including cardiac hypertrophy and fibrosis [18]. In addition, SIRT3 knockout mice were also hypersensitive to cardiac stress induced by transverse aortic constriction (TAC), and to cancer [19]. Simultaneously, Someya *et al*. demonstrated that SIRT3 mediates protective effects of caloric restriction on age-related hearing loss by promoting the mitochondrial antioxidant system through regulation of isocitrate dehydrogenase 2 (Idh2) [20]. Qiu *et al* illustrated that protective effects of caloric restriction (CR) on oxidative stress and damage are diminished in mice lacking SIRT3 [21]. SIRT3 reduces cellular ROS levels dependent on superoxide dismutase 2 (SOD2), a major mitochondrial antioxidant enzyme. Additionally, liver phenotypes appear to be mediated by SIRT3. Recent studies have demonstrated that during fasting, SIRT3-/- mice have diminished fatty acid oxidation, develop a fatty liver, have low levels of ATP production, and show a defect in thermogenesis and hypoglycemia during a cold test [22]. SIRT3 activates hepatic lipid catabolism via deacetylation of LCAD, a central enzyme in the fatty acid oxidation process. Similar phenotypes have been established in mice lacking the mitochondrial enzyme AceCS2—(it is also acetylated by SIRT3 [23]), which suggest that SIRT3 is an important adaptive signal during fasting. Hepatic SIRT3 protein expression increases during fasting [24], therefore both its protein levels and enzymatic activities are elevated during nutrient deprivation.

## SIRT3 activity and NAD levels are suppressed in livers of obese animals

Compared to caloric restriction, relatively little is known about the role of sirtuins and acetylation under conditions of caloric excess. Our recent study [25] adds to the understanding of SIRT3 metabolic function in the context of metabolic stress induced by high fat diet and obesity. We fed a chronic (up to 16 wk) high fat diet (HFD) to mice and demonstrated reduced SIRT3 activity in their livers, a 3-fold decrease in hepatic NAD+ levels, and increased mitochondrial protein oxidation. Using a targeted proteomics approach, we elucidated 193 proteins that were preferentially acetylated in mice on HFD compared to controls, including 11 proteins not previously identified in acetylation studies. HFD led to hyperacetylation of proteins involved in gluconeogenesis, mitochondrial oxidative metabolism, methionine metabolism, liver injury, and ER stress response. In contrast, neither SIRT1 nor histone acetyltransferase (HAT) activities were altered, implicating SIRT3 as a dominant factor contributing to the observed phenotype. Compared to wild-type mice, SIRT3-deficient animals demonstrated an even greater hyperacetylation of gluconeogenic and mitochondrial proteins under HFD conditions. In corroboration with increased acetylation, mice lacking SIRT3 demonstrated a disruption of mitochondrial oxidative phosphorylation complexes II, III, and V. This is the first study to identify acetylation patterns in liver proteins of HFD mice and suggests that SIRT3 and hyperacetylation may play an important role in the regulation of cellular and mitochondrial metabolism induced by high-fat feeding.

The study raises several important questions. Increased mitochondrial protein oxidation in the livers of HFD fed mice is consistent with higher levels of production of mitochondrial ROS under stress conditions [26,27], and suggests that hyperacetylation could play an important role in suppressing function of oxidative stress defense enzymes contributing to HFD-induced liver injury. In mice on HFD, reduced SIRT3 activity was highly correlated with reduced NAD+ levels as the animals became obese. However, the regulatory mechanisms for reduced NAD biosynthesis and distribution of NAD precursors affecting specific sirtuins remain undetermined. Likewise, could the biosynthesis of NAD intermediates and metabolites be effective therapeutic targets and/or reagents for mitochondrial disorders and other diseases? Obesity and fatty liver disease are characterized by the paradoxical accumulation of triglycerides in the insulin-resistant liver [28]. Strong evidence suggests that the maintenance of NAD+ concentration is required for normal mitochondrial fatty acid oxidation [28,29]. It has been shown that pharmacological stimulation of mitochondrial NADH oxidation dramatically promotes beta-oxidation and ameliorates dyslipidemia, adiposity, and fatty liver in obese mice [28]. Nampt overexpression has been shown to maintain cellular NAD levels and thereby stimulates sirtuin activity, resulting in protection of cardiac myocytes from poly(ADP-ribose)polymerase (PARP)-induced cell death during heart failure [30]. Lastly, since diminished mitochondrial function may play a pivotal role in mechanisms regulating insulin resistance, non-alcoholic fatty liver disease, and other metabolic disorders, it remains to be seen whether specific molecular agonists for SIRT3 activity can reverse metabolic disorders such as obesity, type 2 diabetes mellitus and/or inflammatory complications of a high fat diet. If rescuing SIRT3 improves physiological response when challenged with dietary high fat feeding, it will add to the growing body of evidence suggesting that sirtuins might be potential pharmacological targets, not only for extending life span but also for treating metabolic syndrome.

To date, most studies on the biological functions of sirtuins have been conducted in cell culture and mouse models, with studies on possible correlations between human plasma. Therefore, more work needs to be done to elucidate the physiological relevance of sirtuins in normal individuals and in patients with metabolic and other diseases.
